# Mechanical Properties of Fibre Reinforced Polymers under Elevated Temperatures: An Overview

**DOI:** 10.3390/polym12112600

**Published:** 2020-11-05

**Authors:** Milad Bazli, Milad Abolfazli

**Affiliations:** 1 Department of Civil Engineering, Monash University, Clayton 3168, Australia; 2 Department of Civil and Construction Engineering, Swinburne University, Hawthorn 3122, Australia; Mabolfazli@swin.edu.au

**Keywords:** FRP, elevated temperature, fire, post fire, mechanical properties

## Abstract

Fibre-reinforced polymer (FRP) composite is one of the most applicable materials used in civil infrastructures, as it has been proven advantageous in terms of high strength and stiffness to weight ratio and anti-corrosion. The performance of FRP under elevated temperatures has gained significant attention among academia and industry. A comprehensive review on experimental and numerical studies investigating the mechanical performance of FRP composites subjected to elevated temperatures, ranging from ambient to fire condition, is presented in this paper. Over 100 research papers on the mechanical properties of FRP materials including tensile, compressive, flexural and shear strengths and moduli are reviewed. Although they report dispersed data, several interesting conclusions can be drawn from these studies. In general, exposure to elevated temperatures near and above the resin glass transition temperature, *T_g_*, has detrimental effects on the mechanical characteristics of FRP materials. On the other hand, elevated temperatures below *T_g_* can cause low levels of degradation. Discussions are made on degradation mechanisms of different FRP members. This review outlines recommendations for future works. The behaviour of FRP composites under elevated temperatures provides a comprehensive understanding based on the database presented. In addition, a foundation for determining predictive models for FRP materials exposed to elevated temperatures could be laid using the finding that this review presents.

## 1. Introduction

Recently, fibre reinforced polymers (FRPs) have become significantly useful in repair, rehabilitation [1] and strengthening of masonry [2] or reinforced concrete (RC) structural members [3]. FRPs are the principle option for the rehabilitation and strengthening of the existing structures [4]. For example, in many cases, FRP sheets and ropes have been used for strengthening/retrofitting of existing deficient and/or damaged concrete structural members [5,6].

However, multi-story buildings, parking garages, offshore and industrial structures as well as bridges and piers [7,8] are among the numerous potential applications that exist for FRPs. FRPs could be used as either the main member for instance pultruded FRP profiles or replacement to the steel reinforcements in concrete prone to corrosion [9,10].

In recent years, FRP composites have been implemented in various shapes, for instance I-shaped, channels, tubes, etc. [11], reinforcing bars [12], sheets, strips [13], grids and tendons with different types of adhesive amid strengthening or reinforcing structural members such as slabs, beams and columns [14].

Nonetheless, there are concerns with respect to the utilisation of FRP materials in various applications, especially in structures (such as durability of FRP members under aggressive environments, particularly cementitious environment [15,16], and premature debonding failure of FRPs applied in strengthened members [17,18]) where materials need to have satisfactory fire response conduct, maintaining a strategic distance from fire starting, fire spreading and unnecessary smoke creation and spreading. Structural components are additionally expected to introduce adequate imperviousness to fire, so as to offer structural integrity during fire [19,20,21].

**Figure 1:**
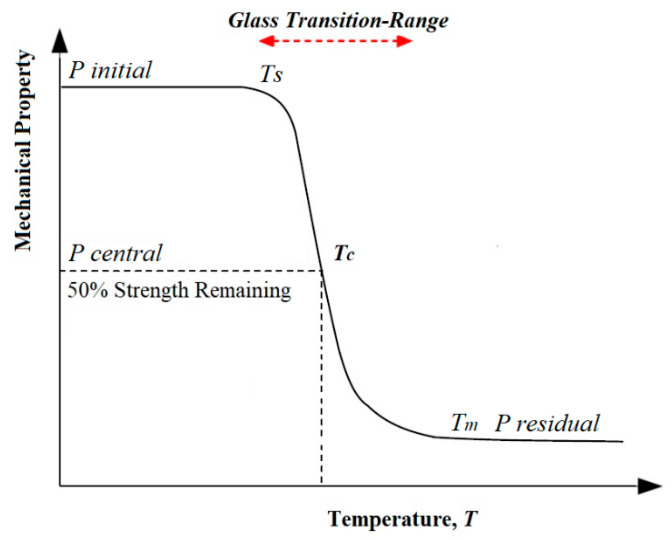
Idealised relationship between FRP mechanical properties and temperature [32].

With this respect, the worries related with the fire performance of FRP materials are genuine [22]. At the point when warmed to mild temperatures, FRP materials mollify and creep, causing a significant decrease of both strength and stiffness [23]. FRP composites arrive at their glass transition temperature *T_g_* typically in the range of 65–120 °C, within which the resin changes from glassy state to rubbery state [24]. Besides, when FRP materials are subjected to high temperatures (300–500 °C), their natural framework deteriorates, delivering heat, smoke, ash and harmful volatiles [25]. Being exposed to this range of elevated temperatures (i.e., resin decomposition temperature *T_d_*) causes the chemical bonds, modular chains of the resin and bonds between the fibres to break [26,27,28,29]. The critical temperature is characterised as the temperature at which the reinforcement loses around half of its strength and can no longer withstand the applied load, according to Wang et al. [30]. Given this definition, the critical temperature (*T_c_*), in this study, it is defined as the temperature at which FRP composite, regardless of the type and configuration, loses 50% of its mechanical strength. Figure 1 shows the idealised relationship between the temperature and the mechanical properties of FRP composite. In this model, no significant mechanical property change is assumed in the initial room temperature until it reaches the softening temperature, *T_s_*. At temperature higher than *T_s_*, the mechanical property decreases to a residual value of $P_{residual}$ with increasing temperature, at which point the composite has reached to the melting temperature, *T_m_*. It is worth mentioning that the region, in which the mechanical property deceases beyond the softening temperature (i.e., changing the state of the rein matrix from brittle state to a rubbery state) is the resin glass transition range. It must also be noted that the literature does not always clearly differentiate between *T_g_* and *T_m_* [31]. Glass transition temperature, *T_g_*, of the resin matrix could be obtained using either differential thermal analysis (DSC) or dynamic mechanical thermal analysis (DMTA) methods. Beyond *T_m_*, the mechanical property decreases gradually [32]. For more information regarding the glass transition, melting and decomposition temperatures, the readers may refer to [31].

A low thermal conductivity of FRP materials is demonstrated notwithstanding such unfavourable behaviour under fire [33]. Thus, they slow down the spread of fire and show a sensible burn-through obstruction, giving a viable boundary against fire and harmful exhaust [34].

Considering the significance of FRP overall performance subjected to extended temperatures, several studies have been completed to research the diverse aspects of FRP behaviour under the effect of temperature [35,36,37,38,39,40,41,42].

The impressive measure of examinations on the presentation of FRP materials under raised temperatures has permitted accomplishing a decent degree of comprehension with respect to the fire/raised temperature response properties of most basic FRP materials utilised in common construction (e.g., FRP bars, profiles, sheets, etc.) [43,44,45,46]. However, such performance varies according to the FRP type and its application in the structure [22,47,48,49,50]. In general, the performance of FRPs in civil applications when subjected to elevated temperatures can be categorised into two groups: (1) when FRP elements are directly subjected to heat flux and fire (e.g., externally strengthened RC beams, concrete filled FRP tubes, bridge piers, etc.) [51,52,53,54,55,56,57,58,59,60]; and (2) when FRP elements are not in direct contact with heat flux and fire (e.g., concrete members reinforced with FRP bars) [61,62,63,64,65].

In this regard, the performance of FRPs are different when they are directly subjected to open flames and abundant oxygen during fire compared to the case of FRPs embedded in concrete. It is worth mentioning that, when FRPs are directly subjecting to fire, the extra heat produced by ignition magnifies the mechanical properties reduction of FRP composites [66,67]. This study reviews the research conducted on the performance of bare FRP members directly exposed to elevated temperatures. The performance of concrete structures strengthened/reinforced with FRP materials under elevated temperatures [68,69,70,71,72] will be reviewed in the future.

The manufacturing technique, type and configuration of fibres and resin and the quality control of the final products are a few variables that affect the material properties of FRP composites [9]. Moreover, based on the different applications of FRPs, different structural performance of the composite structure under elevated temperatures can be observed. With respect to this, Micelli and Nanni [15] called attention to that each FRP composite has specified constituents and production methods; consequently, the ends drawn for one material are not relevant to others. However, more reliable conclusions may be drawn when larger number of studies are considered and compared with each other.

This review presents the findings of more than 100 experimental and numerical studies in order to foundationally explore the behaviour of FRP composites exposed to raised temperatures for giving a reliable information for the field. In this paper, the damage mechanisms and machinal performance of FRP bars under elevated temperatures are first discussed. Then, the results of the performance of FRP laminates and sheets, as well as pultruded GFRP profiles with different cross-section configurations, are presented. Eventually, suggestions for future work are given, and the conclusions are drawn. This enormous data base is expected to give a far-reaching comprehension to the field and a strong establishment for future work.

It is worth mentioning that the main focus of the literature, and consequently this review paper, is on glass and carbon fibres, with more focus on glass compared to carbon. The research on the behaviour of other fibres, such as basalt, is limited and thus the conclusions drawn are less reliable for those types of composites.

## 2. FRP Reinforcing Bars

Recently, interest for the utilisation of FRP reinforcement in concrete structures as an option in contrast to conventional material, specifically steel reinforcement, has expanded [73,74]. FRP reinforcement is advantageous over steel as it is highly corrosion resistant and enjoys a high strength to weight ratio [62,75]. Although the impact of elevated temperatures on FRP reinforced structures could be a design necessity, this matter has not yet been studied comprehensively [76,77].

### 2.1. Degradation Mechanism

Based on the literature, the degradation mechanism of FRP reinforcing bars under elevated temperature can be divided into four groups. (1) When subjecting to elevated temperature below glass transition temperature, *T_g_*: in this case, the resin matrix surface will remain rough similar to unconditioned sample. Within this temperature range, some micro cracks will be observed in the resin matrix. (2) When subjecting to elevated temperate near and slightly above *T_g_*: in this case, the resin matrix starts to soften, revealing the fibres positions. This confirms that exposure to temperatures around *T_g_* will affect the resin matrix and result reduction in tensile strength of FRP reinforcing bars. (3) When subjecting to elevated temperatures above *T_g_* and below *T_d_*: in this case, the surface of the resin matrix becomes almost smooth, and, due to the resin softening, fibres become more visible (i.e., significant fibre/resin debonding). This explains further tensile properties reduction of FRP bars compared to the unconditioned samples or the samples subjected to conditions 1 and 2. (4) When subjecting to elevated temperatures near and above *T_d_*: in this case, the resin matrix surrounding the fibres will almost be lost, and thus fibres become more visible. This confirms the fibre/resin debonding and consequently significant tensile properties reduction of FRP bars. It is worth mentioning that these ranges of elevated temperatures do not affect the fibres properties.

**Figure 2:**
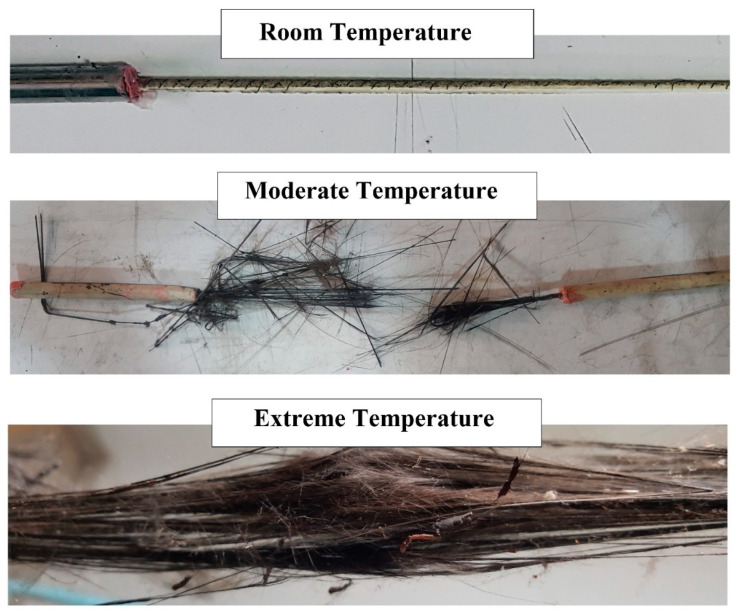
Typical failure modes of FRP reinforcing bars after exposure to elevated temperatures.

Figure 2 shows the typical failure modes of FRP bars after exposure to elevated temperatures. As is seen at room temperature, fibres still bound together, and the failure mode is the group fibres rupture. However, after subjecting to elevated temperature, due to the resin softening and consequently fibre/resin interface debonding, fibres are separated and fail individually. Finally, at very high temperatures, resin is burnt and fibres are exposed and visible. In Figure 2, moderate and extreme temperatures represent temperatures near *T_g_* and *T_d_*, respectively.

### 2.2. Mechanical Properties

There are numerous studies focused on the mechanical properties of FRP reinforcing bars under elevated temperatures. Both experimental tests and numerical analyses have been conducted on a wide range of FRP bars (i.e., different fibre types, resins, bars diameter and surface configurations) and elevated temperatures (i.e., ranging from ambient to fire condition).

Kumahara et al. [35] extensively studied various forms of FRP reinforcement bars subjected to elevated temperatures between 60 and 400 °C. GFRP reinforcing bars with two different types of resin, vinyl ester and polyphenylene sulphide (PPS), were tested in addition to carbon and aramid reinforced composites. PPS is a thermoplastic resin with resistance to heat. It was shown that bars with PPS demonstrate different performance in comparison to the GFRP bars with vinyl ester resin. When warmed to 250 and 400 °C, bars with vinyl ester lost 40% and 60% of their primary strength, respectively. Their tensile strength also started to diminish at temperatures over 60 °C. On the other hand, the GFRP bars with PPS did not experience the ill effects of the heating up to 250 °C. The better exhibition of the last was accepted to be a direct result of the better heat-resistance qualities of PPS sap contrasted with vinyl ester. There are not many detailed examinations on the elastic modulus of GFRP bars during presence to raised temperatures. They likewise considered the modulus of elasticity of two kinds of GFRP bars with either a vinyl ester or a thermoplastic, polyphenylene sulphide (PPS) resin. For the primary sort of bar, the modulus of elasticity at 350 °C diminished to 40% of the room temperature while the subsequent kind gave no indication of debasement in modulus.

Sayed-Ahmed and Shrive [78] studied CFRP tendons and reported that after 24 h at 200 and 300 °C, the surface of CFRP tendons had become darker, indicating some resin loss. Some of the fibres on the surface became loose after 24-h exposure at 400 °C. On the other hand, being exposed to 500 °C caused resin to evaporate mainly within the first hour of exposure, which reduces the tendon to a bundle of loose fibres.

The behaviour of newly developed GFRP bars under extreme heat was investigated by Nause [79]. It was reported that up to a temperature of about 450 °C, there is a linear reduction in the tensile strength. A reduction of approximately 35% of the primary tensile strength of the bars was measured at this temperature. The tensile strength experienced a significant reduction after exposure to 450 °C. A complete degradation of the resin matrix was considered the main reason for this significant reduction.

An experimental study of the mechanical characteristics of GFRP and Carbon FRP (CFRP) bars at raised temperatures was conducted by Wang et al. [27]. The stress-strain relationships of FRP bars stayed almost linear at elevated temperatures until failure based on the results obtained from the experiments. A gradual reduction in the tensile strength of FRP bars at elevated temperatures, however, was observed at an almost linear rate to zero at about 500 °C. Although until 300–400 °C their elastic modulus remained consistent, there was a sharp drop in the elastic modulus after this temperature.

GFRP bar samples at various elevated temperatures (23–315 °C) were tested in research conducted by Robert and Benmokrane [67]. Some adverse effects of high temperature on tensile strength of the GPRP bars were reported throughout the experiments. It was concluded that, at high temperatures, the mechanical characteristics, especially the stiffness and the strength of the composites, were diminished considerably at temperatures higher than the glass transition temperature of the polymer matrix. The fall in the tensile strength of the bars at about 315 °C was about 53% of the primary tensile strength.

The effect of elevated temperatures on the mechanical characteristics of various FRP bars was conducted in a study by Hamad et al. [26]. They reported that at a critical temperature of 325 °C, there was about 55% and 30% loss in their tensile strength and elastic modulus, respectively.

To study the residual tensile properties of GFRP bars, Ellis et al. [80] heated the bars up to 400 °C and then cooled them down to room temperature. According to the results, GFRP bars retained 83% of their room tensile capacity after exposure to 400 °C and cooling to the room temperature.

Hajiloo et al. [9] investigated the effects of resin content and thermal characteristics on tensile behaviour of GFRP bars under elevated temperatures varying from room temperature to 500 °C. Based on their test results, GFRP bars retained only about 25% of their tensile capacity at 400 °C.

The effect of exposure duration on tensile properties of GFRP bars after subjecting to elevated temperatures was investigated by Alsayed et al. [81]. Strength reductions of 9.7–41.9% were reported, while no considerable reduction in tensile modulus was observed. In addition, the strength deterioration was found to be almost linear with respect to the temperature and exposure duration.

Wang and Zha [66] reported 22% and 67% tensile strength reductions at 120 and 500 °C, respectively, after studying GFRP bars under elevated temperatures.

Ashrafi et al. [82] investigated the effects of bar diameter, fibre and resin type, fibre to resin ratio and thermal properties on the tensile performance of different FRP bars subjected to elevated temperatures. They reported almost the same critical temperatures for GFRP and CFRP bars (i.e., 300–450 °C): the larger diameter bars showed the higher critical temperature. In another study by Ashrafi et al. [83], the tensile strength of GFRP bars with different diameters subjected to elevated temperatures (15–300 °C) was investigated. As expected, no considerable changes were observed in tensile strength at temperatures up to 60 °C. However, at higher temperatures, close to *T_g_*, a sudden drop was observed in tensile properties due to the resin softening. At extreme temperatures (i.e., higher than 200 °C), more than 65% strength reduction was observed due to the significant resin degradation and consequently resin/fibre debonding.

In another study by Wang and kodur [30], the GFRP and CFRP bars lost 50% of their capacity at 325 and 250 °C, respectively. With respect to the elastic modules, less than 10% reduction was observed below 350 °C. However, at 350 °C, GFRP and CFRP bars reached to their critical temperature (i.e., 50% reduction in elastic modulus).

Similarly, Ozkal et al. [84] tested GFRP bars in tension at 23–800 °C and found the critical temperature of the bars to be 300 °C.

Yu and Kodur [85] studied the mechanical properties of CFRP rods under elevated temperatures and reported no significant mechanical properties degradation below 200 °C. However, at temperature higher than 330 °C, about 50% reduction in ultimate tensile strength was observed.

Zhou et al. conducted both transient and steady state tensile tests to study the tensile properties of CFRP tendons under elevated temperatures [86]. Based on the test results, the critical temperature of CFRP tendons, at which tendons lose 50% of their room temperature capacity, were 324 and 341 °C in steady state and transient state, respectively.

Maranan et al. [76] performed experimental tests to investigate the flexural behaviour of FRP bars when subjected to elevated temperatures: 100 and 150 °C. As expected, severe reductions in both flexural strength and stiffness of the bars were reported when the testing temperature reaches the *T_g_*. The flexural strength and stiffness were decreased about 53% and 30%, respectively, at 100 °C. The corresponding values at 150 °C, were 94% and 66%, respectively. In addition, it was concluded that the greater is the bar’s diameter, the better is the flexural performance, indicating the importance of size effect consideration in studying the thermal-mechanical performance of FRP bars.

**Figure 3:**
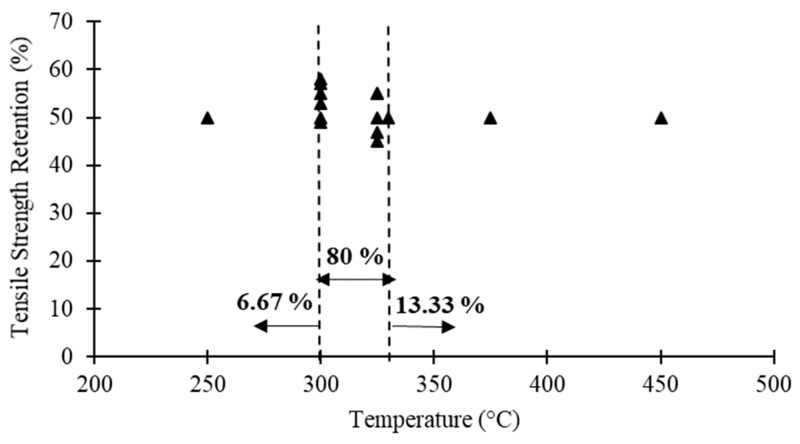
FRP bars tensile strength versus critical temperature reported in the literature.

Table 1 compares the steady state results of several studies in terms of the critical temperature (i.e., temperature corresponding to a strength reduction of about 50%), elastic modulus retention corresponding to the critical temperature and the mechanical properties at extreme temperatures (i.e., the maximum temperature applied in each study). In addition, Figure 3 shows FRP bars tensile strength versus critical temperature reported in these studies. Although different materials and testing parameters have been used in each study, the following general conclusions can be drawn based on Table 1 and Figure 3: (1) Regardless of the material type (i.e., fibre and resin type), the critical temperature of FRP reinforcing bars is generally between 300–330 °C. (2) At extreme temperatures less than 450 °C, FRP bars are still able to carry some loads. (3) At temperatures higher than 450 °C, FRP bars almost lose their tensile strength (i.e., retention less than 20%). (4) The tensile elastic modulus is significantly less affected by elevated temperatures compared to the tensile strength. (5) GFRP and BFRP bars perform relatively better than CFRP bars under elevated temperatures (except the results in [82]).

Researchers have used these experimental results to propose predicting models of FRP bars tensile properties under elevated temperatures. Some of these models are presented in Section 3.2.2 together with FRP laminates tensile properties. Table 2 also lists the transient state tensile test results of GFRP and CFRP bars under different stress ratios, which could be used for modelling of such bars in RC structures under fire condition.

## 3. FRP Laminates

Considering several advantages of FRP sheets and laminates, they are mainly used to enhance the mechanical properties, including the strength, stiffness and ductility of concrete structures such as beams, columns, and slabs [87]. In these applications, high temperature behaviour and fire performance of composites are important issues [88,89]. Considerable research has focused on the mechanical properties of FRP laminates at elevated temperatures [44,90,91]. In this section, the studies carried out on different mechanical properties of FRP laminates, including tensile, flexural and compressive properties, are reviewed.

### 3.1. Degradation Mechanism

Similar to FRP reinforcing bars, different failure mechanisms will occur in FRP laminates when subjected to different elevated temperature ranges: (1) When the temperature is below *T_g_*, the failure mode is brittle, and fibres are still surrounded by the resin matrix. In this case, the temperature has insignificant effect on the tensile behaviour and failure mode of FRP laminates/sheets. (2) When the temperature reaches the resin *T_g_*, the resin softens and causes the separate fibres fracture [92]. In other words, the laminates fail due to the resin softening and gasification following by fibre rupture. In this case, considerable mechanical properties degradation will occur, and part of the epoxy will also be lost. (3) When the temperature reaches *T_d_*, almost no resin will be left since the resin reaches to its self-ignition temperature. This will let the specimen bend freely after the tensile test. (4) Finally, at very extreme temperatures, the resin matrix will completely burn, and a considerable fraction of fibres will oxidise [93].

**Figure 4:**
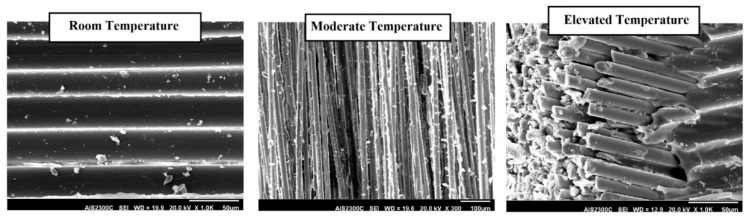
SEM images of FRP laminates after exposure to elevated temperatures.

**Figure 5:**
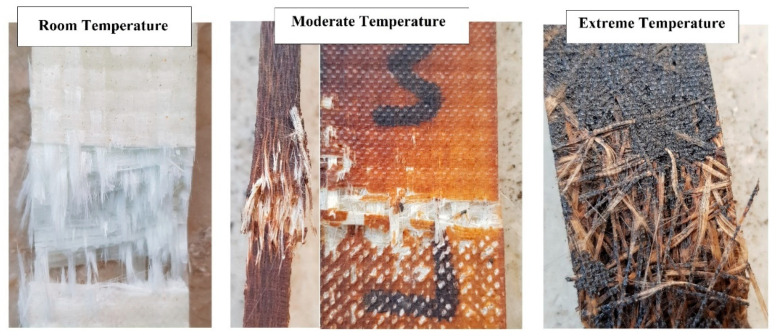
Typical failure modes of FRP laminates after exposure to elevated temperatures.

Figure 4 illustrates the degradation mechanisms of FRP composites through scanning electron microscopy (SEM) analysis. As is seen, by increasing the temperature, the resin matrix starts to degrade (i.e., softening, cracking and eventually burning off) resulting in the fibres becoming visible and separated. Figure 5 also shows the typical failure modes of FRP laminates after exposure to elevated temperatures. As is seen, similar to FRP bars, fibres are bound together before reaching the critical temperature, while fibres separation, layers delamination and resin softening and ignition occur by increasing the temperature.

### 3.2. Mechanical Properties

#### 3.2.1. Tensile Properties

There are numerous studies focused on the mechanical tensile properties of FRP laminates/sheets under elevated temperatures. Both experimental tests and numerical analyses have been conducted on a wide range of FRP laminates (i.e., different fibre types, resins, laminate/sheet thicknesses and orientations) and elevated temperatures (i.e., ranging from ambient to fire condition). Using the test results, several prediction models have been proposed by researchers to predict the mechanical properties of different FRP laminates under elevated temperatures.

The effect of laminate thickness and fibre orientation on the tensile properties of GFRP laminates was investigated by Jafari et al. [94]. According to their test results, laminates with unidirectional fibres showed the best performance, maintaining almost 40% of their tensile strength capacity at 550 °C. However, laminates with woven and chopped strand mat fibres lost all their tensile strength, respectively, at 550 and 400 °C. In another study on similar laminates, Ashrafi et al. [95] investigated exposure duration effect and cooling phase. They concluded that although the duration of heat exposure is an effective factor, the degradation of GFRP laminates tensile strength is irreversible after 20 min.

The results of the study conducted by Jarrah et al. [96] on the tensile properties of GFRP and CFRP sheets under elevated temperatures up to 600 °C show a significant strength reduction up to 87% and 67%, respectively, for GFRP and CFRP sheets at 600 °C.

Wang et al. [93] studied both steady and transient states performance of pultruded CFRP plates under tension loads at temperatures ranging from ambient to 700 °C. It was shown that the plates lose almost half of their tensile strength at 300 °C, while they maintain 7% of their capacity at 700 °C.

After conducting tensile tests on CFRP and hybrid glass/basalt and carbon FRP sheets under elevated temperatures up to 200 °C, Cao et al. [97] showed 40% strength reduction. They also observed similar performance for CFRP sheets and hybrid FRP sheets under elevated temperatures.

Cao et al. [98] also studied tensile behaviour of CFRP sheets when subjected to temperatures between 20 and 120 °C. Similar to previous research, they showed a stable behaviour for CFRP sheets at temperatures lower than *T_g_* and a rapid drop in tensile strength when the temperature reaches *T_g_*.

In an experimental study by Lu et al. [92], the high temperature effect on the tensile behaviour of pultruded BFRP plates and basalt fibre roving was investigated. Tensile strength and elastic modulus reductions of 37.5% and 31% were reported, respectively, for BFRP plates at 200 °C, while the corresponding values were 8.3% and 9.7%, respectively, for basalt fibre roving.

Gibson et al. [99] reported 80% strength reduction of woven GFRP composites at 300 °C. Kumarasamy et al. [100] investigated the tensile performance of GFRP laminated manufactured using the wet hand lay-up at high temperatures of 25–80 °C. A strength reduction of about 65% was observed for samples with polyester resin after exposure to 80 °C.

Hawileh et al. [101] carried out an experimental study on basalt, carbon and their hybrid laminates to investigate their tensile properties when exposed to elevated temperatures up to 250 °C. It was observed that all laminates lost up to 90% of their tensile strength when exposed to 250 °C. The same authors [102] also investigated the similar properties of glass, carbon and their hybrid laminates. The reported that the hybrid laminates under elevated temperatures show better performance in comparison to the glass and carbon laminates. For instance, elastic modulus reductions of about 28%, 26% and 9%, respectively, were obtained after exposure to 250 °C.

Foster and Bisby [103] performed a series of experimental tests on carbon and glass sheets to investigate the effect of elevated temperatures (up to 400 °C) on their tensile properties. It was reported that glass laminates underwent a major strength reduction at 200 °C, while no significant reduction was observed in carbon samples at temperatures up to 300 °C.

Wu et al. [104] showed that after 2-h exposure to 200 °C, hybrid basalt-carbon sheet can retain almost 60% of its residual strength.

Sim et al. [105] performed an experimental study on the tensile properties of different types of glass, carbon and basalt sheets after 2-h exposure to high temperatures ranging from 100 to 1200 °C and 24-h cooling at room temperature. Based on their reported results, no considerable strength reduction was observed for all types of FRPs at temperatures below 200 °C, while, at temperatures above 200 °C, a significant strength reduction was shown for CFRP and GFRP. However, only 10% strength reduction was observed for basalt fibres at 600 °C.

GFRP laminates were tested in tension at temperatures ranging from ambient to 80 °C with various strain rates by Reis et al. [106]. It was shown that strain rate is an important factor in tensile strength of GFRPs, while it does not affect the elastic modulus.

Shekarchi et al. [107] studied the ultimate tensile strength of GFRP and CFRP laminates subjected to elevated temperatures ranging from 25 to 500 °C and reported tensile strength reductions of 83% and 70%, respectively, for GFRP and CFRP laminates after exposure to 500 °C. The corresponding reductions were about 48% and 39%, respectively, at 200 °C

In the study of Aydin [36], laminates cut from pultruded GFRP box profiles were tested at different temperatures up to 200 °C under tension. The results showed tensile strength reductions about 6%, 18%, 28%, 30%, 38%, 40% and 47% at 50, 75, 100, 125, 150, 175 and 200 °C, respectively, compared to reference samples tested at room temperature.

By testing GFRP tensile coupons under transient and steady-state thermal conditions at temperatures from ambient to 200 °C, Chowdhury et al. [32] reported that GFRP samples lose almost half of their tensile strength near *T_g_*. However, they retain 40% of their room temperature capacity when subjected to 200 °C.

In another study, Correia et al. [108] experimentally and analytically studied the tensile properties of pultruded GFRP laminates under elevated temperatures ranging from 20 to 250 °C. It was found that, although the tensile strength is significantly reduced due to the elevated temperatures, specimens could still retain 54% of their tensile capacity at 220 °C.

Bai and Keller [109] tested pultruded GFRP laminates under elevated temperatures ranging from room temperature to 220 °C and reported about 80% strength reduction at 220 °C compared to the laminates tested at ambient temperature. It is worth noting that at temperatures above 100 °C, the premature failure at grips affected the test results.

**Figure 6:**
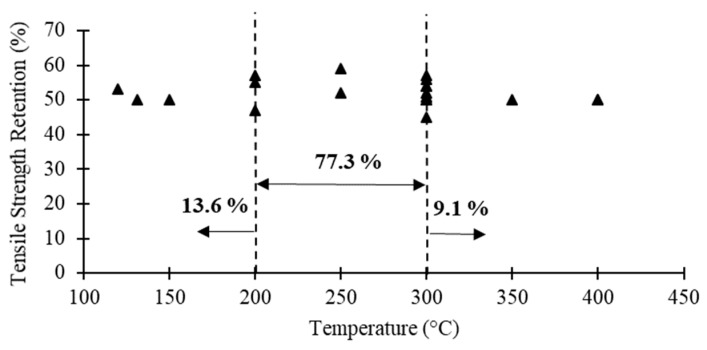
FRP laminates tensile strength versus critical temperature reported in the literature.

Table 3 compares the steady state results of several studies on FRP laminates/sheets in terms of the critical temperature, tensile elastic modulus retention corresponding to the critical temperature and tensile properties at extreme temperatures. Figure 6 also presents the FRP laminates tensile strength versus critical temperature reported in the literature. According to Table 3 and Figure 6, the following conclusion can be listed: (1) Compared to the FRP reinforcing bars, the data of laminates under elevated temperatures are more scattered; different fabrication methods used to construct FRP sheets/laminates could be the main reason for this difference. (2) Regardless of the material type (i.e., fibre and resin type), the critical temperature of FRP laminates is generally between 200–300 °C (except limited studies). (3) The critical temperature of the laminate is relatively lower than that of the FRP bars. The fibres to resin fraction ratio (i.e., typically the fibre to resin ratio is greater in FRP bars compared to the FRP laminates) is the main reason for this observation. (4) BFRP samples perform better than GFRP and CFRP samples under elevated temperatures. (5) Similar to FRP bars, the tensile elastic modulus is generally less affected by elevated temperatures compared to the tensile strength. (6) At extreme temperatures over 400 °C, FRP laminates/sheets almost lose all their tensile strength (i.e., reduction values from 68% to 94%). 

#### 3.2.2. Tensile Properties Predicting Models

Given the tensile properties of FRP composites as an important factor in designing composite structures under elevated temperatures [102], using experimental test data, several researchers have proposed models for predicting the tensile properties of such material when subjected to elevated temperatures. With this regard, the primary model was proposed by Gibson et al. [110] considering the temperature as the only parameter affecting the tensile properties retention of FRP composites. Many other researchers used the primary model of Gibson et al. and proposed similar models based on their experimental data [62,85,93,101]. However, by using linear Bayesian regression, limited studies have also considered the effects of different factors, such as exposure time and composite thickness [94,95,111,112].

Gibson et al. [110] proposed the following general models and noted that both models are capable of predicting the mechanical properties of FRP composites under elevated temperatures:

(1) $$P\left(T\right)=\frac{P_{u}+P_{R}}{2}-\frac{P_{u}-P_{R}}{2}\;\mathrm{erf}(k\left(T-T^{\prime }\right))$$

(2) $$P\left(T\right)=\frac{P_{u}+P_{R}}{2}-\frac{P_{u}-P_{R}}{2}\;\tan h\left(k\left(T-T^{\prime }\right)\right)$$

where P(T). is the specific property of the composite, $P_{u}$ is the unrelaxed property value (i.e., property at low temperature), $P_{R}$ is the relaxed property value (i.e., property at high temperature), *k* is the distribution constant and $T^{\prime }$ is the determined glass transition temperature.

Using the primary model of Gibson et al. [110], Yu and Kodur [85] predicted the tensile properties of CFRP pultruded strips using Equations (3) and (4), respectively, for tensile strength and elastic modulus:

(3) R(T)=0.56−0.44tanh(0.0052(T−305))

(4) R(E)=0.51−0.49tanh(0.0035(T−340))

Saafi et al. [62] proposed Equations (5) and (6) to predict respectively the GFRP bars tensile strength and elastic modulus based on the data presented in the study of Blontrok et al. [77]

(5) R(T)=1−0.0025T 0≤T≤400 °C

(6) $$R\left(E\right)=\{\begin{array}{ll} 1 & 0\leq T\leq 100\;{}^{\circ}C\\ 1.25-0.0025T & 100\;{}^{\circ}C\leq T\leq 300\;{}^{\circ}C\\ 2-0.005T & 300\;{}^{\circ}C\leq T\leq 400\;{}^{\circ}C \end{array}$$

The relation between the temperature and tensile strength of CFRP laminates was predicted by Wang et al. [93] using Equation (7):

(7) $$R\;\left(T\right)=\{\begin{array}{ll} 1-\frac{{\left(T-22\right)}^{0.9}}{200} & 22\;{}^{\circ}C\leq T\leq 150\;{}^{\circ}C\\ 0.59-\frac{{\left(T-150\right)}^{0.7}}{490} & 150\;{}^{\circ}C\leq T\leq 420\;{}^{\circ}C\\ 0.48-\frac{{\left(T-420\right)}^{1.8}}{76000} & 420\;{}^{\circ}C\leq T\leq 706\;{}^{\circ}C \end{array}$$

Hawileh et al. [101] proposed Equations (8) and (9) to predict, respectively, the tensile strength and modulus of BFRP sheets after exposure to elevated temperatures:

(8) R(T)=0.795−0.205tanh(0.075(T−190.58)

(9) R(E)=0.86−0.140tanh(0.035(T−163.24))

Jafari et al. [94] performed linear Bayesian regression analysis to derive predicting models for tensile strength of GFRP laminates with different sample thicknesses and fibres orientations:

(10) $$R\;\left(T\right)=\{\begin{array}{ll} 1 & 24\;{}^{\circ}C\leq T\leq 45\;{}^{\circ}C\\ a\;\left(\frac{1}{T^{3}}\right)+b\;\left({\left(Log\left(t\right)\right)}^{0.333}\right)+c & 45\;{}^{\circ}C\leq T\leq 500\;{}^{\circ}C \end{array}$$

where *T* (° K) and *t* (mm) are the temperature and thickness of the laminate, respectively, and a–c are the regression constants with respect to the fibres orientations [94]. Ashrafi et al. [95] also took into account the exposure time effect and presented Equations (11) and (12) to predict the tensile strength and elastic modulus of the same laminates, respectively:

(11) $$R\;\left(T\right)=a\;\left(\frac{1}{T^{3}}\right)+b\;\left(\frac{1}{{\left(Log\left(\frac{t_{1}}{6}\right)\right)}^{0.5}}\right)-c\;\left(\frac{1}{{\left(Log\left(t_{2}\right)\right)}^{0.5}}\right)+d$$

(12) $$R\;\left(E\right)=-a\;{\left(T\right)}^{4}+b\;\left(\frac{1}{{\left(Log\left(\frac{t_{1}}{6}\right)\right)}^{0.5}}\right)-c\;\left(\frac{1}{{\left(Log\left(t_{2}\right)\right)}^{0.5}}\right)+d$$

where *t*_1_ (min) and *t*_2_ (mm) are the exposure time and laminate thickness, respectively, and a–d are the regression constants with respect to the fibres ordinations [95].

**Figure 7:**
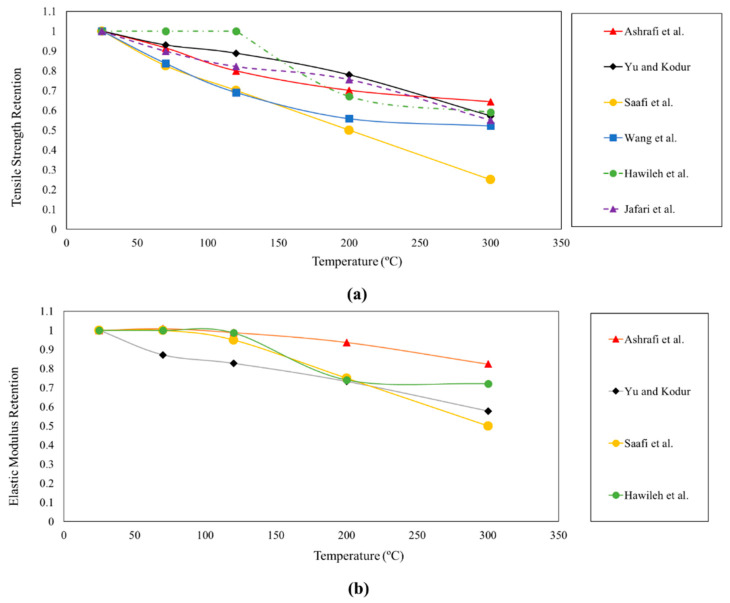
Comparison of predicting models: (**a**) tensile strength retention vs. temperature; and (**b**) tensile elastic modulus vs. temperature.

To compare the proposed models, Figure 7 shows the results predicted by some of the presented models related to the tensile performance of composites with continuous fibres subjected to elevated temperatures up to 300 °C. As expected, due to the several parameters, such as test protocols, fibre type, material properties and cross-section configuration, different trends are observed. However, a general conclusion is that the elastic modulus of FRP composites is less affected by elevated temperatures than the tensile strength.

#### 3.2.3. Flexural Properties

Some researchers also studied the flexural and interlaminar shear strengths of FRP laminates/sheets when exposed to elevated temperatures. With this regard, Ningyun and Evans [113], as one of the early studies in this area, conducted short beam tests to investigate the flexural properties of FRP laminates (graphite fibre/thermoplastic matrix composites) under elevated temperatures up to 300 °C. They showed that the laminates behave linearly at lower temperatures and eventually fail due to delamination fracture. However, at higher temperatures (i.e., 200 °C), laminates experience some nonlinearity in their load-displacement curve. Finally, at extreme temperature (i.e., 300 °C), a considerable nonlinearity was observed at low loads and laminates lost about 75% of the room strength at this temperature.

Bazli et al. [112] investigated the effects of laminates thickness and fibres orientation on the flexural strength of vacuum-infused GFRP laminates under elevated temperatures ranging from room temperature to 300 °C. It was observed that thinner laminates are more vulnerable in flexure than thicker laminates. In addition, it was reported that laminates with continuous unidirectional fibres show better performance compared to laminates with woven and chopped strand mat fibres. However, regardless of the laminate thickness and fibres orientation, all specimens lost almost all their flexural strength capacity at 300 °C.

Recently, Shekarchi et al. [107] studied the flexural performance of GFRP and CFRP laminates after exposure to temperatures ranging from 25 to 350 °C. Significant strength reduction (i.e., 89% for CFRP and 93% for GFRP laminates) at 350 °C was reported. The corresponding strength reductions were about 50% for CFRP and 40% for GFRP laminates at 200 °C.

Flexural behaviour of GFRP skins and GFRP skins and a phenolic core sandwich were studied by Manalo et al. [114] under temperatures ranging from room temperature to 180 °C. According to their test results, all samples retained more than 80% of their flexural strengths at 80 °C, while the corresponding values at 150 °C for GFRP skins and sandwich beams were 40% and 19%, respectively.

Recently, Vieira et al. [115] conducted experimental tests on the residual shear and flexural performance of pultruded GFRP laminates subjected to elevated temperatures up to 320 °C. The results of bending tests were scattered; however, in contrast to other research studies, generally the flexural and interlaminar shear strengths did not show considerable decrease (maximum of 25% and 12% reductions for bending and shear samples, respectively).

Schmidt et al. [116] studied two pultruded GFRP laminates with different resins (isophthalic polyester and phenolic resins) in flexure when subjected to elevated temperatures. It was concluded that laminates with phenolic resin performs significantly better compared to the laminates with isophthalic polyester.

**Figure 8:**
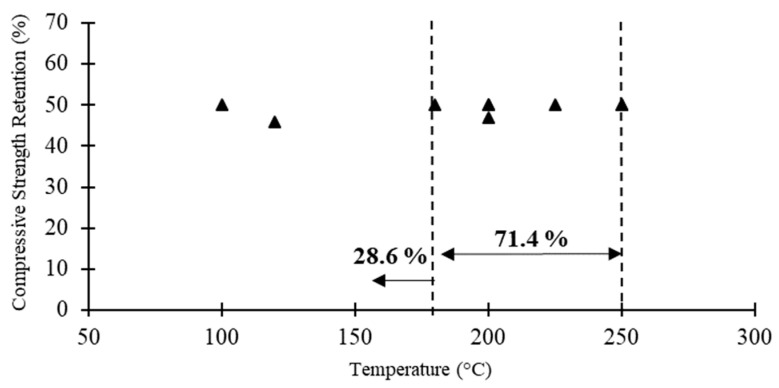
FRP laminates flexural strength versus critical temperature reported in the literature.

Table 4 compares the steady state flexural and interlaminar shear test results of several studies on FRP laminates/sheets in terms of the critical temperature and the flexural properties at extreme temperatures. The data regarding the FRP laminates flexural strength versus critical temperature reported in the literature are also shown in Figure 8. Considering the data in Table 4 and Figure 8, one can conclude the followings: (1) Regardless of the material type (i.e., fibre and resin type), the critical temperature of FRP laminates in flexure is generally between 180–250 °C. (2) The critical temperature related to flexural strength of FRP laminates is considerably lower than that of the tensile strength. The main reason for this difference is the different failure modes typically occur in tensile and flexural tests. It is generally known that resin matrix degradation affects the strength of FRP composites flexural properties, especially interlaminar shear strength compared to the tensile properties. (3) The performance of laminates with unidirectional fibres are better than that of laminates with woven and chopped strand mat fibres. (4) The maximum temperature that FRP laminates can withstand during bending tests is less than 400 °C, which is significantly lower than that of tensile tests. This confirms the fact that, at extreme temperatures, during tensile tests, fibres still carry some loads, while the resin/fibre interface degradation under elevated temperatures will allow the early failure of FRP laminates under bending.

#### 3.2.4. Compressive Properties

A limited number of studies have also investigated the compressive properties of FRP laminates when exposed to elevated temperatures. However, more experimental and numerical studies are needed to better understand the compressive performance (i.e., local and global buckling behaviour) of FRP laminates under compressive loading.

Gibson et al. [99] studied the compressive properties of polypropylene composites with woven glass fibres when subjected to fire condition (i.e., 50 kW/m^2^ heat flux). They reported that almost all compressive strength of the specimen (more than 90%) was lost at 140 °C.

Asaro et al. [117] tested FRP composite panels subjected to combined thermal (IMO A.754 or UL 1709 flames) and compressive loading and indicated that the exposure time and temperature affect the degradation of composite materials, and consequently their strength and failure mode during fire.

Bai and Keller [118] carried out compressive tests on slender GFRP laminates under elevated temperatures up to 180 °C. and reported an experimental buckling load reduction up to 60% at 180 °C.

The failure of FRP laminates subjected to one-sided heating from a fire and compressive loads was predicted by Summers et al. [119]. In a similar study, Feih et al. [120] modelled the compressive strength of polymer laminates when exposed to one-sided radiant heating by fire.

Table 5 compares the steady state compression test results of three studies on FRP laminates in terms of the critical temperature and the compressive strength at extreme temperatures. By comparing the data presented in Table 5 with those listed in Table 2 and Table 4, one can simply conclude that the strength of FRP laminates subjected to elevated temperatures is significantly more vulnerable in compression compared to tension and bending. The critical temperature is between 80 and 140 °C, and reductions between 60% and 90% at temperatures less than 200 °C confirm this conclusion.

## 4. FRP Profiles

Currently, there are many applications for FRP profiles in construction. Piping, pedestrian bridge decks, off-shore rigs, panel walls, trains, trail decks, waste treatment plants, high performance automobiles, aircrafts, marine crafts and thermo-electrical plants are among these applications [10,121,122,123,124,125]. Due to this wide range of applications, recently, design guides, such as ASCE [126], EUR27666 [127] and CECS [128] have been established for pultruded FRP composites in many countries.

Given that FRP profiles will be subjected to elevated temperatures and open fires due to their usage in different sites [8], their mechanical and stability performance under elevated temperatures is a great concern [129,130]. Therefore, many studies have addressed the performance of different FRP profiles, including beams and columns, under elevated temperatures. However, due to the vulnerability and insufficient knowledge of pultruded FRP profiles when subjected to elevated temperatures, the adoption of load-bearing members in civil structures are still low [131]. Therefore, to widely use such members in civil structures, more attention and data are needed in this area [62]. In this paper, studies carried out on pultruded GFRP profiles are reviewed in two groups: beams and columns.

### 4.1. Beam

There are several studies in the literature that investigated the performance of FRP structures as the load bearing members under elevated temperatures, and, according to the FRP temperature-dependent thermo-physical properties, models have been proposed to predict the thermal responses of such structures under elevated temperatures [20,29].

Ludwig et al. [132] investigated the fire resistance performance of 1.5-m long pultruded I-section (IPE120 and IPE160) GFRP beams. All beam surfaces were subjected to ISO 834 fire and 30% of the beam failure load was applied at midspan. IPE120 and IPE160 profile beams subjected to fire failed after only 1.45 and 2.25 min, respectively, due to upper flange local buckling followed by web buckling. It was also reported that the average temperatures of failure of specimens were 120 and 155 °C for IPE120 and IPE160, respectively.

The fire resistance behaviour of pultruded GFRP box beams (1.5 m long and cross-section of 100 mm with wall thickness of 8 mm) was studied by Correia et al. [131]. Four-point bending tests with loading corresponding to midspan deflection equal to L/400 were carried out. The bottom surface of the beam was subjected to ISO 834 fire in order to simulate the embedded beam in a floor slab. Based on the test results, the beam failed after 38 min.

In another study, Correia et al. [108] reported that the shear strength of GFRP beam decreased up to 89% after exposure to 250 °C in comparison to the beam tested at ambient temperature.

Wijayawardane et al. [133] also studied the pultruded GFRP I-beams flexural properties under elevated temperatures. It was reported that beams lose only 15% of their flexural strength after exposure to elevated temperatures up to 60 °C. On the other hand, they showed that both stiffness and strength deterioration increase by increasing the temperature and the failure mode was related to the wall’s delamination.

Based on the study of the behaviour of pultruded GFRP beams under fire, Morgado et al. [134], concluded that the number of surfaces being exposed to fire is an important factor. According to their test results, an unprotected beam with three sides being exposed to fire condition showed a significantly higher fire strength reduction compared to the beam with only one side being exposed to fire.

Mouritz [135] studied the flexural properties of different GFRP profiles when subjected to open fire. Three types of resin were used: polyester, epoxy and phenolic. As expected, it was shown that the higher are the heat flux and exposure time, the greater is the depth of the profiles char layers, and thus the greater is the mechanical properties degradation. They also showed that chopped glass composites with different resins lose 50% of their flexural strength and modulus when subjected to 50 kW/m^2^ heat flux for an exposure time of 75–150 s.

Table 6 presents three-point bending (3PB) and four-point bending (4PB) test results of pultruded GFRP beams under ISO 834 fire. Considering the data in Table 6, the following conclusions can be made: (1) when only one side of the beam is subjected to fire, FRP beams can resist the service load for more than 30 min; and (2) the fire resistance duration of unprotected FRP beams decreases significantly when the number of sites that are exposed to fire condition increases (e.g., from 36 to 8 min). This confirms that using fire protection will significantly increase the fire resistance capacity of GFRP beams when subjected to fire condition [136].

### 4.2. Columns

**Figure 9:**
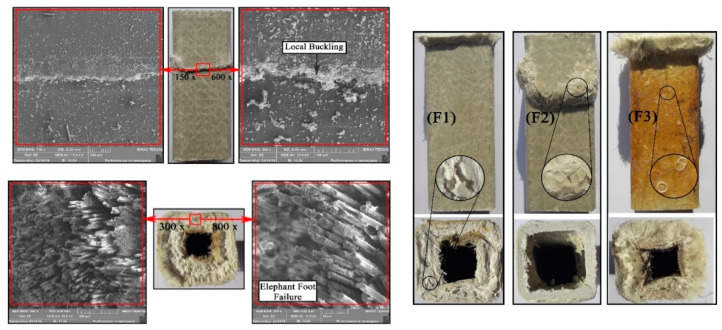
Typical failure modes of compressive pultruded GFRP profiles [137].

According to previous research studies [108], it is generally accepted that GFRP profiles when subjected to elevated temperatures are much more vulnerable in compression than in tension. It is also shown that, in the case of exposing the bottom flange of the profiles to extreme temperatures, even higher than the resin decomposition temperature (*T_d_*) and for a long period, the profile never fails in tension. Generally, failure occurs at lower temperatures in compression and/or shear (the failure mostly occurs at the web under the applied load and/or at the top flange at midspan [130]). Therefore, understanding the compressive behaviour of pultruded profiles when exposed to elevated temperatures is a key. Local buckling and failure due to the brooming effect at a sample ends are the most common failure modes observed during a compression test. Figure 9 shows the failure modes and SEM images of compressive pultruded GFRP profiles under elevated temperatures. According to the authors of [137], failure modes of pultruded GFRP profiles under elevated temperatures are classified into three groups. As shown in Figure 9, failure mode F1 is due to the brooming effect (elephant foot buckling in some references). Regardless of the temperature, this failure mode is one of the typical failure modes of FRP profiles under compression. However, at temperatures near and above *T_g_*, resin crippling and interlaminar shear failure were also reported as dominant failure modes (i.e., F2). Finally, at extreme temperatures (i.e., near *T_d_*), since resin almost loses its load transferring capacity, early resin crippling together with fibres buckling (sever brooming (i.e., F3)) occur at very low loads.

Wang and Wong [138] tested pultruded GFRP channel columns in compression at temperatures ranging from 20 to 120 °C. The effect of column height (ranging from 0.5 to 1.5 m) and the rotational restraints about major and minor axes were investigated. It was shown that the columns tested about the minor axis failed due to the global buckling towards the web and lost up to 66% of their compressive strength at 120 °C. Regarding the columns tested about the major axis, short columns failed due to the combination of material crushing and buckling, while the longer columns failed in either local or global buckling. This type of columns showed up to 38% compressive strength reduction at 120 °C. Short channel columns (100 × 30 × 4 mm and 30 mm long) were also tested at a wider range of temperatures (i.e., 20–250 °C) by Wang et al. [131,138] and up to 92% compressive strength reduction was reported at 250 °C.

Bai and Keller [109] studied the compressive behaviour of GFRP columns (cross section of 40 mm × 3 mm and of 300 mm long) subjected to elevated temperatures ranging from ambient to 220 °C. They observed dramatic compressive strength reductions around the resin glass transition temperature with the maximum strength reduction up to 90% at 220 °C.

Wong et al. [131] tested pultruded GFRP C-shaped columns in compression at temperatures varying between 20 and 250 °C. It was concluded that profiles will retain most of their compressive strength when subjected to temperatures below *T_g_*, while significant compressive strength reduction was observed at temperatures above *T_g_*, reaching almost 85% at 250 °C.

The effects of profile cross-section configuration and slenderness on the compressive behaviour of pultruded GFRP profiles under elevated temperatures ranging from ambient to 400 °C were studied by Khaneghahi et al. [137]. It was observed that GFRP profiles lose 50% of their compressive strength when exposed to temperatures close to the resin *T_g_* (i.e., temperatures above 90 °C). Moreover, the specimens lost almost all their compressive capacity at temperatures above 120 °C.

A similar conclusion was derived by Correia et al. [108] stating that the pultruded GFRP boxes experience considerable compressive strength reductions under elevated temperatures, which reaches up to 95% after exposure to 250 °C.

Russo et al. [19] conducted compressive tests to investigate the residual strength of pultruded GFRP boxes under different temperature cycles. The samples were firstly subjected to 50, 100, 150 and 200 °C and then cooled down to room temperature. According to their results, only 14% strength reduction was observed after applying significant thermal load cycles. It is worth mentioning that the maximum strength reduction for the highest temperature was 25%.

Aydin [36] conducted compression tests on pultruded GFRP box sections subjected to elevated temperatures up to 200 °C. Strength reductions of 18%, 25%, 67%, 78%, 88.5% and 94% were reported at 50, 75, 100, 125, 150 and 175 °C, respectively, compared to the samples tested at 25 °C. Moreover, it was shown that GFRP profiles lose almost all their compressive strength when exposed to 200 °C.

Najafabadi et al. [111] studied the mechanical properties of various GFRP profiles, including I- and box-shaped sections after exposure to elevated temperatures (25–550 °C). Their test results showed that, at temperatures lower than 200 °C, most of the mechanical properties are retained, while, at temperatures higher than 200 °C, up to 30% reductions in mechanical properties were observed. It was also shown that the compressive strength reduction of GFRP profiles is lower than that of flexural strength. It is worth mentioning that the elevated temperature in this study was applied in the absence of ambient oxygen.

**Figure 10:**
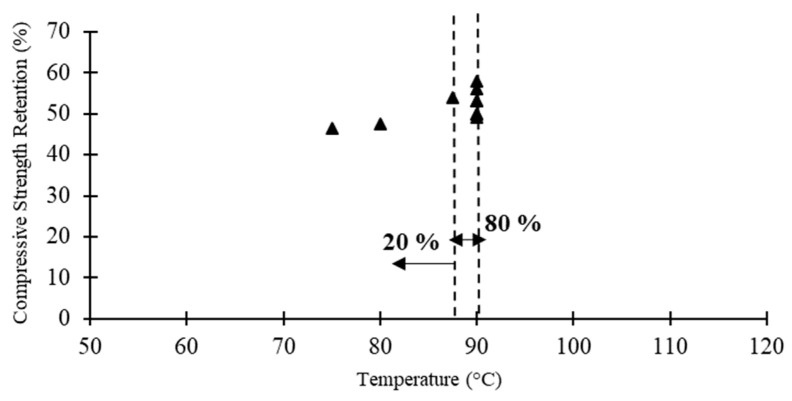
Pultrude GFRP profiles compressive strength versus critical temperature reported in the literature.

Table 7 compares the steady state compression test results of pultruded GFRP profiles under elevated temperatures in terms of the critical temperature, compressive elastic modulus corresponding to the critical temperature and the compressive properties at extreme temperatures. The data regarding the Pultrude GFRP profiles compressive strength versus critical temperature reported in the literature are also shown in Figure 10. From the results in Table 7 and Figure 10, the following conclusions can be drawn: (1) GFRP profiles reach the critical temperature (i.e., temperature corresponding to 50% compressive strength reduction) significantly earlier than other mechanical properties (i.e., *T_c_* of 87–90 °C); (2) before reaching *T_d_*, pultruded profiles almost lose all of their compressive strength; and (3) similar to other mechanical properties, the compressive elastic modulus is less affected under elevated temperatures compared to the compressive strength.

## 5. Recommendations for Future Work

Based on the studies reviewed in this paper, the following recommendations are proposed for future work:

(1) More experimental results under real fire condition (e.g., ISO 834) are needed to understand the effect of different factors, on fire resistance of FRP composites.
(2) The data related to the effects of fibres content and orientations on performance of FRP composites under elevated temperatures exist but are very limited, thus further work is needed in this area.
(3) More experimental tests on BFRP composites are needed to better understand the effects of different factors on performance of such new composites under elevated temperatures.
(4) To investigate whether a structure that was exposed to high temperatures can still be used or damages can be repaired, more studies are required on the post-fire properties of FRP composites.
(5) Finally, by using the available data and proposed models, the current codes and guidelines should be developed to include specific procedures for the fire design of FRP composites.

## 6. Conclusions

A comprehensive review is presented out on the mechanical performance of FRP composites, including reinforcing bars, laminates/sheets and pultruded profiles, subjected to elevated temperatures. Based on the findings from these studies, the following main remarks can be concluded:

(1) When subjected to elevated temperatures below glass transition temperature, *T_g_*, the resin matrix will not be significantly affected (i.e., some micro cracks may occur) and the surface of the resin matrix will remain rough and similar to the unconditioned sample. In this case, no dramatic strength and stiffness reductions of FRP composites occur.
(2) When FRP composites reach their glass transition temperature *T_g_*, the resin changes from glassy state to rubbery state. In this case, FRP materials soften and creep, causing a considerable reduction of both strength and stiffness.
(3) When FRP materials are exposed to temperatures around resin decomposition temperature, their organic matrix decomposes, releasing heat, smoke, soot and toxic volatiles. Exposure to such range of elevated temperatures (e.g., 300–500 °C) leads to breaking of the chemical bonds, modular chains of the resin and bonds between the fibres. The ignition and combustion of the composite occur at higher temperatures.
(4) The critical temperature (i.e., temperature corresponding to 50% strength reduction) is generally 300–330 °C for FRP reinforcing bars, 200–300 °C for laminates in tension, 180–250 °C for laminates in bending and 87–90 °C for pultruded GFRP profiles in compression.
(5) FRP composites fail in compression and interlaminar shear at significantly lower loads and exposure temperature than in tension and flexure.
(6) Elastic modulus of FRP composites is less affected by elevated temperatures compared to the corresponding strength values. This is mainly due to the fact that the elastic modulus of FRP composites is more related to the elastic modulus of fibres than resin.

## Figures and Tables

**Table 1:** FRP bars tensile properties after exposure to elevated temperatures (steady state tensile tests).

Study	Fibre Type	Resin Type	Bar Size	*T_g_*	Critical Temperature (T1)	Strength Retention at T1	Modulus Retention at T1	Maximum Test Temperature (T2)	Strength Retention at T2	Modulus Retention at T2
[27,30]	Carbon	Polyester	9.5	NA	250	50%	80%	600	6%	35%
	Glass	Polyester	9.5	NA	325	50%	90%	500	16%	NA
[26]	Carbon	Vinyl ester	10	NA	325	45%	68%	450	10%	37%
	Glass	Polyester	10	NA	325	55%	79%	375	9%	52%
	Basalt	Epoxy	10	NA	325	55%	79%	375	13%	47%
[82]	Carbon	Epoxy	5	110	300	55%	NA	450	35%	NA
	Glass	Epoxy	4	110	300	50%	NA	450	29%	NA
		Epoxy	10	110	450	50%	NA	450	50%	NA
[80]	Glass	Vinyl ester	19	NA	NA	NA		400	83%	83%
[84]	Glass	Epoxy	9	NA	375	50%	75%	500	10%	7%
[86]	Carbon	Epoxy	8	126	300	53%	NA	500	17%	NA
[83]	Glass	Epoxy	10	95	300	57%	NA	300	57%	NA
[67]	Glass	Vinyl ester	12.7	113	325	47%	NA	325	47%	NA
[81]	Glass	Vinyl ester	12	NA	300	58%	No change	300	58%	No change
[9]	Glass	Vinyl ester	16	110	300	49%	75%	400	41%	75%
[85]	Carbon	NA	6.4	80	330	50%	60%	600	8%	20%

**Table 2:** FRP bars subjected to elevated temperatures (transient state tensile tests).

Study	Fibre Type	Resin Type	Bar Size	*T_g_*	Stress Ratio (%)	Loading Rate (°C/min)	Failure Temperature (°C)
[9]	Glass	Vinyl ester	16	110	22	5	518
					35		425
					47		327
					53		193
					59		157
[86]	Carbon	Epoxy	8	126	28.8	20	468
					38.5		431
					48.1		366
					57.7		239
					67.3		183

**Table 3:** FRP laminates/sheets tensile properties after exposure to elevated temperatures (steady state tensile tests).

Study	Fibre Type	Fibres Orientation	Resin Type	Specimen Dimensions (in mm)	*T_g_*	Critical Temperature (T1)	Strength Retention at T1	Modulus Retention at T1	Maximum Test Temperature (T2)	Strength Retention at T2	Modulus Retention at T2
[97]	Carbon	Unidirectional	epoxy	400 × 200 (1 layer of fibre)	38	NA	NA	NA	200	68%	NA
[102]	Carbon	Unidirectional	epoxy	250 × 40 × 0.348	NA	300	54%	39%	300	54%	39%
	Glass	Unidirectional	epoxy	250 × 40 × 0.352	NA	300	57%	70%	300	57%	70%
[101]	Carbon	Unidirectional	epoxy	250 × 40 × 0.99	85	131	50%	20%	250	19%	9%
	Basalt	Unidirectional	epoxy	250 × 40 × 0.95	85	250	59%	73%	250	59%	73%
[94]	Glass	Unidirectional	epoxy	Dog-bone (600 × 20 × 2)	70	300	56%	NA	550	17%	NA
	Glass	Woven	epoxy	Dog-bone (600 × 20 × 2)	70	300	51%	NA	400	8%	NA
	Glass	Chopped strand mat	epoxy	Dog-bone (600 × 20 ×2)	70	60–80	50%	NA	250	13%	NA
[95]	Glass	Unidirectional	epoxy	300 × 20 × 5	70	300	45%	82%	300	45%	82%
	Glass	Woven	epoxy	300 × 20 × 5	70	200	55%	87%	300	35%	68%
	Glass	Chopped strand mat	epoxy	300 × 20 × 5	70	120	53%	89%	300	6%	24%
[96]	Carbon	Woven	epoxy	500 × 30 (1 layer of fabric)	60	300	50%	NA	600	32%	NA
	Glass	Woven	epoxy	500 × 30 (1 layer of fabric)	60	400	50%	NA	600	13%	NA
[93]	Carbon	Unidirectional	epoxy	600 × 26 × 1.4	60	300	54%	NA	700	6%	NA
[92]	Glass	Unidirectional	epoxy	15 (width) × 1.27	167	200	57%	80%	200	57%	80%
	Basalt	Unidirectional	epoxy	16 (width) × 1.27	167	NA	NA	NA	200	63%	69%
[99]	Glass	Unidirectional	Polypropylene	Dog-bone (300 × 15 × 12)	NA	150	50%	NA	300	25%	NA
[98]	Carbon	Unidirectional	epoxy	250 × 10 × 0.111	45	NA	NA	NA	120	70%	NA
[107]	Carbon	Unidirectional	epoxy	600 × 25 × 2.5	100	300	52%	NA	500	30%	NA
	Glass	Unidirectional	epoxy	600 × 25 × 2.5	100	250	52%	NA	500	17%	NA
[36]	Glass	Unidirectional	Polyester	200 × 20 × 4	NA	200	47%	NA	200	47%	NA
[32]	Glass	Unidirectional	epoxy	735 × 38 × 2.6	75	75	48%	77%	200	46%	81%
[85]	Carbon	Unidirectional	NA	13.5 (wide) × 4.5 (thickness)	80	300	50%	53%	600	11%	NA
[103]	Carbon	Unidirectional	epoxy	1 (thickness)	78	400	50%	NA	400	50%	NA
	Glass	Unidirectional	epoxy	1.3 (thickness)	78	350	50%	NA	400	20%	NA

**Table 4:** FRP laminates/sheets flexural and interlaminar shear properties after exposure to elevated temperatures.

Study	Fibre Type	Fibres Orientation	Resin Type	Laminate Dimensions	*T_g_*	Critical Temperature (T1)	Strength Retention at T1	Maximum Temperature (T2)	Strength Retention at T2
[112]	Glass	Unidirectional	epoxy	70 × 18 × 5	70	225	50%	300	8%
	Glass	Woven	epoxy	70 × 18 × 5	70	200	50%	300	4%
	Glass	Chopped strand mat	epoxy	70 × 18 × 5	70	200	47%	300	4%
[92]	Basalt	Unidirectional	epoxy	7.8 × 2.6 × 1.27	167	100	50%	200	10%
[107]	Carbon	Unidirectional	epoxy	100 × 25 × 2.5	100	250	50%	350	11%
	Glass	Unidirectional	epoxy	100 × 25 × 2.5	100	200	50%	350	7%
[113]	Carbon	Unidirectional	Polyetheretherketone	12 × 30 × 2	NA	180	50%	300	25%
[114]	Glass	Woven and chopped strand mat	phenolic	220 × 50 × 5	NA	120	46%	180	52%
[116]	Glass	Unidirectional	Polyester	135 × 25 × 6.9	NA	250	50%	250	50%
		Unidirectional	Phenolic	90.5 × 25 × 4.2	NA	NA	NA	250	80%
[115]	Glass	Unidirectional	Polyester	240 × 25 × 9.5	100	NA	NA	320	No reduction
		Unidirectional	Vinyl ester	155 × 16 × 6	113	NA	NA	320	No reduction
		Unidirectional	Phenolic	240 × 25 × 9.5	120	NA	NA	320	No reduction
		Unidirectional	Polyester	95 × 47.5 × 9.5	100	NA	NA	270	No reduction
		Unidirectional	Vinyl ester	60 × 30 × 6	113	NA	NA	270	83%
		Unidirectional	Phenolic	95 × 47.5 × 9.5	120	NA	NA	270	93%

**Table 5:** FRP laminates/sheets compressive properties after exposure to elevated temperatures.

Study	Fibre Type	Fibres Orientation	Resin Type	Laminate Dimensions	*T_g_*	Critical Temperature (T1)	Strength Retention at T1	Maximum Temperature (T2)	Strength Retention at T2
[118]	Glass	Unidirectional	polyester	400 × 48 × 12	155	140	57%	180	40%
[99]	Glass	Woven	Polypropylene	125 × 105 × 12	NA	80	50%	140	7%
[120]	Glass	Woven	Vinyl ester	100 × 100 × 9	120	100	45%	180	7%

**Table 6:** Pultruded GFRP beams performance subjected to ISO 834 fire [129].

Study	Cross Section	Span/Height (m)	Number of Sides	Load	Fire Resistance (min)
[130]	Square tubular (h = 100 mm, tf = tw = 8 mm)	1.5	1	L/400 (4PB)	38
[134]	Square tubular (h = 100 mm, tf = tw = 8 mm)	1.3	1	L/400 (4PB)	36
	Square tubular (h = 100 mm, tf = tw = 8 mm)	1.3	3	L/400 (4PB)	8
	Square tubular (h = 100 mm, tf = tw = 8 mm)	1.3	1	L/250 (4PB)	31
[132]	IPE 120	1.5	4	10 kN (3PB)	1.45
	IPE 160	1.5	4	10 kN (3PB)	2.25

**Table 7:** Pultruded GFRP (polyester resin) profiles compressive properties after exposure to elevated temperatures.

Study	Profile Type (Length × min Thickness)	*T_g_*	Critical Temperature (T1)	Compressive Strength Retention at T1	Modulus Retention at T1	Maximum Temperature (T2)	Compressive Strength Retention at T2	Modulus Retention at T2
[137]	I-shaped (4.3 mm)	95	90	53%	NA	400	2%	NA
	Channel (5 mm)		90	53%	NA	400	2%	NA
	Box (3 mm)		90	50%	NA	400	5%	NA
	Angle (6 mm)		90	49%	NA	400	3%	NA
[138]	Channel (500 mm × 5 mm)	NA	90	56%	78%	120	40%	65%
	Channel (900 mm × 5 mm)		NA	NA	NA	120	62%	84%
	Channel (1350 mm × 5 mm)		NA	NA	NA	120	67%	66%
[131]	Channel (30 mm × 4 mm)	NA	60–90	63%–30%	NA	250	8%	NA
[139]	Channel (400 mm × 4 mm)	NA	90	58%	70%	250	8%	30%
[36]	Box (74 mm × 3 mm)	NA	75–100	75%–33%	NA	175	6%	NA
[108]	I-shaped (50 mm × 6 mm)	136	90	44%	NA	250	5%	NA
[109]	Tube (300 mm × 3 mm)	110	60–100	65%–30%	NA	220	10%	NA
